# Long-Term Follow-Up of Patients after Acute Kidney Injury: Patterns of Renal Functional Recovery

**DOI:** 10.1371/journal.pone.0036388

**Published:** 2012-05-04

**Authors:** Etienne Macedo, Dirce M. T. Zanetta, Regina C. R. M. Abdulkader

**Affiliations:** 1 Discipline of Nephrology, School of Medicine, University of São Paulo, São Paulo, Brazil; 2 Department of Epidemiology, School of Public Health, University of São Paulo, São Paulo, Brazil; University of Sao Paulo Medical School, Brazil

## Abstract

**Background and Objectives:**

Patients who survive acute kidney injury (AKI), especially those with partial renal recovery, present a higher long-term mortality risk. However, there is no consensus on the best time to assess renal function after an episode of acute kidney injury or agreement on the definition of renal recovery. In addition, only limited data regarding predictors of recovery are available.

**Design, Setting, Participants, & Measurements:**

From 1984 to 2009, 84 adult survivors of acute kidney injury were followed by the same nephrologist (RCRMA) for a median time of 4.1 years. Patients were seen at least once each year after discharge until end stage renal disease (ESRD) or death. In each consultation serum creatinine was measured and glomerular filtration rate estimated. Renal recovery was defined as a glomerular filtration rate value ≥60 mL/min/1.73 m2. A multiple logistic regression was performed to evaluate factors independently associated with renal recovery.

**Results:**

The median length of follow-up was 50 months (30–90 months). All patients had stabilized their glomerular filtration rates by 18 months and 83% of them stabilized earlier: up to 12 months. Renal recovery occurred in 16 patients (19%) at discharge and in 54 (64%) by 18 months. Six patients died and four patients progressed to ESRD during the follow up period. Age (OR 1.09, p<0.0001) and serum creatinine at hospital discharge (OR 2.48, p = 0.007) were independent factors associated with non renal recovery. The acute kidney injury severity, evaluated by peak serum creatinine and need for dialysis, was not associated with non renal recovery.

**Conclusions:**

Renal recovery must be evaluated no earlier than one year after an acute kidney injury episode. Nephrology referral should be considered mainly for older patients and those with elevated serum creatinine at hospital discharge.

## Introduction

The incidence of acute kidney injury (AKI) is increasing in hospitalized patients [1]–[3]. Although previous studies have shown a high rate of renal recovery among survivors, most of them defined recovery as dialysis independence at hospital discharge. The lack of a consistent definition for recovery has resulted in differing prevalence of renal recovery across the literature [4]–[9]. The Acute Dialysis Quality Initiative (ADQI) consensus defines complete renal recovery as return to baseline classification within the RIFLE criteria and partial recovery as a change in RIFLE status in patient free of dialysis [10]. In spite of this, few studies have evaluated renal recovery in the context of this recommendation. Ali et al found that 68% of their population had full renal recovery and 5% had partial recovery based on the return of serum creatinine to its baseline value [1]. The majority of other studies have included only patients who required dialysis and defined recovery as dialysis independence at discharge [6], [11], [12]. Rates of renal recovery in these studies have varied widely from 36% to 99% [13]–[15].

Although only a minority of patients will require dialysis at hospital discharge, patients with partial renal recovery are at increased risk for ESRD. Recent epidemiologic studies have shown that the proportion of CKD population presenting AKI as an accelerating factor for ESRD is underestimated [13], [16]. The factors associated with renal recovery or with progression to ESRD after an episode of AKI are not well established. Despite increasing recognition that renal recovery after AKI is an important outcome, surprisingly little data defining this outcome is available. To help clarify the factors associated with renal recovery we studied 84 patients who had AKI and described the pattern of their renal recovery.

## Methods

The Research Ethics Committee of Hospital das Clínicas, School of Medicine, University of São Paulo, (CAPPesq number 0592/06) approved the study and waived informed consent as there was no intervention, the study was retrospective and used only a data bank, where confidentiality was guaranteed.

From July 1984 to October 2008, patients who had an episode of AKI were referred to nephrology follow up after hospital discharge by attending physicians. A nephrologist (RCRMA) was responsible for follow up of each patient during the observation period. The patient was usually seen 1 to 2 weeks after the discharge and each 3 months afterwards until serum creatinine (sCr) stabilized. After the initial observation period, the patient was seen every 6 to 12 months until death, ESRD or appropriate referral because he or she had reached stable GFR. During nephrology follow up, the causes associated with the AKI episode, the peak sCr, renal function at hospital discharge and pre-existing co-morbidities were retrospectively recorded from the medical chart. Serum creatinine was measured at each consultation and GFR was estimated by abbreviated Modification of Diet Renal Disease (MDRD) equation. In this analysis we included patients 16 years old or older, with a follow-up period of more than 18 months and with more than three sCr measurements during this minimum period. We excluded patients with a baseline sCr higher than 3 mg/dL, and those with AKI associated with glomerulonephritis, vasculitis, multiple myeloma or urinary obstruction. AKI was defined as an increase in sCr of more than 50% from reference sCr, or a sCr more than 2 mg/dL in patients with unknown baseline sCr and without any evidence of previous renal disease. Reference sCr was considered the value obtained within one year before hospital admission. [17], [18] Renal recovery was defined as the absence of CKD defined as a GFR <60 mL/min/1.73 m^2^ at 18 months after the hospital discharge [19] For patients with known reference sCr, we also defined renal recovery as a GFR value more than 90% of reference GFR. [20] We assessed renal recovery at hospital discharge, up to 18 months after discharge and at the time of the best GFR achieved during entire follow-up [18], [21]. We classified patients in 4 groups based on the best GFR achieved up to 18 months after AKI: G1 (n = 26) - GFR ≥90; G2 (n = 28) - GFR ≥60 and <90; G3 (n = 24) - GFR ≥30 and <60 and G4 (n = 6) - GFR ≥15 and <30 mL/min per 1.73 m^2^. GFR was also analyzed at 24, 36, 48 and 60 months. When there was more than one value of GFR within these periods, we considered the mean as representative of the period. The GFR of each patient was last checked in July 2010 as well as his/her status relative to the nephrology clinic: was the patient active; was the patient referred to another hospital,; did the patient abandon the follow up in the hospital; had the patient died or been referred to chronic dialysis.

Statistical analysis: Continuous variables are presented as mean±SD or median (25^th^ – 75^th^ percentiles) and were compared using one-way ANOVA or Kruskal-Wallis test according with the Gaussian distribution. Categoric variables are presented as absolute number and percentage and were compared by Chi-square test. Comparison between reference GFR and best GFR up to 18 months was performed using paired t-test. P<0.05 was considered significant. These statistical analyses were performed using GraphPad 4.00 for Windows (GraphPad Software, San Diego, CA, USA). Multiple variable logistic regression was performed to evaluate partial recovery defined as being in G3 or G4 (reference: G1 and G2 grouped). We used a forward method with the likelihood ratio for covariate entry and tested the models using the Hosmer and Lemeshow test. Variables candidates were: age, hypertension, diabetes, the etiology of AKI (nephrotoxicity, hypovolemia), and sCr at hospital discharge (P<0.05 in univariate analysis.). For all categoric variables reference was “no”. The results of logistic regression are presented as OR and lower and upper 95% confidence interval. Logistic regression was performed using SPSS software 18.0.

## Results

**Figure 1:**
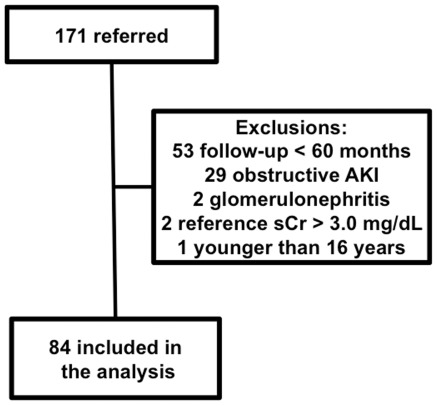
Flowchart of patients included in the analysis. AKI: acute kidney injury; sCr: serum creatinine.

During the study period, 171 patients with an episode of AKI were referred to RCRMA’s office; of those, 87 were excluded from the study as shown in figure 1. The main cause for exclusion was a follow-up period lower than 18 months for 53 patients. The median length of follow-up was 50 months (30–90 months). Seventy patients (83%) had stabilized their GFR up to 12 months and the remaining ones up to 18 months. Table 1 shows the main characteristics of the patients. Age increased from group G1 to G4. The frequency of co-morbidities was similar among all groups except for hypertension, which was more frequent in G3 (p = 0.003). Seven patients had only one functional kidney but this was not associated with a worse recovery (p = 0.583).

**Table 1:** Main characteristics of the four groups: demographic and clinical data.

	All N = 84	G1 N = 26	G2 N = 28	G3 N = 24	G4 N = 6	P1	P2
Age (years)	49 (36–63)	33 (29–39)	49 (37–52)	63 (52–68)	68 (42–72)	<0.001	<0.001
Gender (male)	56 (66.7%)	19 (73.1%)	17 (60.7%)	16 (66.7%)	4 (66.7%)	0.818	1.000
Hypertension	30 (40%)	5 (20.8%)	8 (36.4%)	16 (69.6%)	1 (16.7%)	0.003	0.014
	N = 75	N = 24	N = 22	N = 23	N = 6		
Diabetes mellitus	10 (13%)	3 (13%)	1 (3.7%)	5 (23.8%)1	1 (16.7%)	0.230	0.151
	N = 77	N = 23	N = 27	N = 21	N = 6		
Malignancy	11 (13.1%)	1 (3.8%)	3 (10.7%)	5 (20.8%)	2 (33.3%)	0.137	0.048
One functional kidney	7 (8.7%)	2 (7.7%)	1 (3.8%)	3 (13.6%)	1 (16.7%)	0.583	0.232
	N = 80	N = 26	N = 26	N = 22	N = 6		

G1 - GFR ≥90; G2 - GFR ≥60 and <90; G3 - GFR ≥30 and <60; G4 - GFR ≥15 and <30 mL/min per 1.73 m^2^. Classification based on the best glomerular filtration rate (GFR) achieved up to 18 months after discharge. P1: comparison among all the four groups; P2: comparison between G1+G2 and G3+G4 grouped.

The AKI characteristics are presented in table 2. The peak sCr during AKI was not significantly different among the groups (G1–G4), P = 0.169. The need for dialysis was more frequent in G2 than in G1 and G3 (P = 0.016 and P = 0.005, respectively). Oliguria also tended (p = 0.053) to be more frequent in G2; however we must point out that there were 50% of missing data for this variable. All patients in G4 had been admitted in clinical services. SCr at discharge was progressively higher from G1 to G4 (p<0.001).

**Table 2:** Characteristics of the acute kidney injury episode.

	All N = 84	G1 N = 26	G2 N = 28	G3 N = 24	G4 N = 6	P1	P2
Oliguria	20 (45.5%)	5 (38.5%)	11 (73.3%)	3 (25%)	1 (33.3%)	0.053	0.060
	N = 44	N = 13	N = 15	N = 12	N = 4		
Admission to ICU	48 (64.9%)	14 (58.3%)	18 (78.3%)	13 (61.9%)	3 (50%)	0.403	0.460
	N = 74	N = 24	N = 23	N = 21	N = 6		
Clinical/surgical/obstetric service	55/22/7	17/5/4	22/4/2	10/13/1	6/0/0	0.008	0.021
Allergic acute nephritis	8 (9.5%)	3 (1.5%)	2 (7.1%)	3 (12.5%)	0 (0%)	0.756	1.000
Sepsis	34 (40.5%)	8 (30.8%)	13 (46.4%)	10 (41.7%)	3 (50%)	0.643	0.817
Leptospirosis	11 (13.1%)	6 (23.1%)	5 (17.9%)	0 (0%)	0 (0%)	0.061	0.006
Nephrotoxicity	16 (19.0%)	3 (11.5%)	4 (14.3%)	6 (25%)	3 (50%)	0.130	0.081
Hypovolemia	34 (40.5%)	8 (30.8%)	8 (28.6%)	16 (66.7%)	2 (33.3%)	0.022	0.010
Low cardiac output	9 (10.7%)	1 (3.8%)	2 (7.1%)	5 (20.8%)	1 (16.7%)	0.217	0.063
LOS (days)	23 (14–38)	20 (13–41)	29 (12–38)	22 (14–31)	65 (12–85)	0.532	0.835
	N = 78	N = 22	N = 28	N = 22	N = 6		
Dialysis	49 (60.5%)	13 (50%)	22 (84.6%)	10 (43.5%)	4 (66.7%)	0.015	0.103
	N = 81	N = 26	N = 26	N = 23	N = 6		
Peak sCr at AKI (mg/dL)	7.5 (5.8–12.0)	8.8 (6.0–11.7)	9.6 (6.4–14.7)	5.9 (4.6–10.8)	8.0 (5.4–15.2)	0.169	0.059
sCr at discharge (mg/dL)	2.1 (1.6–2.9)	1.3 (1.0–2.2)	1.9 (1.7–2.7)7	2.3 (1.9–3.3)	4.1 (3.5–5.5)	<0.001	<0.001
	N = 80	N = 23	N = 27	N = 24	N = 6		

LOS: length of stay from acute kidney injury diagnosis to hospital discharge; sCr : serum creatinine; AKI: acute kidney injury; P1: comparison among all the four groups; P2: comparison between G1+G2 and G3+G4 grouped. G1 - GFR ≥90; G2 - GFR ≥60and<90; G3 - GFR ≥30 and <60; G4 - GFR ≥15 and <30 mL/min per 1.73 m^2^. Classification based on the best glomerular filtration rate (GFR) achieved up to 18 months after discharge.

**Figure 2:**
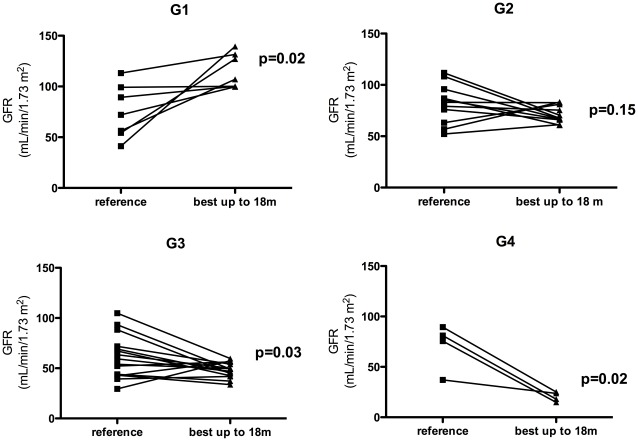
Progression of the glomerular filtration rate from reference to 60 months after acute kidney injury for patients in groups G1 to G4. GFR: estimated glomerular filtration rate; m: months; ref: reference; AKI: lowest value during the acute kidney injury episode; discharge: hospital discharge. ×: patient progressed to ESRD. Δ: patient died.

The GFR progression of all groups is presented in figure 2 and table 3. The best GFR observed during the follow-up was achieved earlier in G1 patients (4.7 months [2.1–7.0 months]) than in G2 (29.5 months [11.0–52.6 months]) and G3 patients (24.8 months [8.8–67.5 months]). At the end of the follow-up, GFR was higher in G1 than in G3 and G4 (p<0.001).

**Table 3:** Best and final glomerular filtration rate and time of assessment during the follow-up period.

	All N = 84	G1 N = 26	G2 N = 28	G3 N = 24	G4 N = 6	P1	P2
Best GFR during the follow-up(mL/min per 1.73 m^2^)	80 (56–105)	116 (102–130)	81 (72–86)	49 (43–61)	24 (19–31)	<0.001	<0.001
Time to best GFR (months)	11.5 (5.4–40.7)	4.7 (2.1–7.0)	29.5 (11.0–52.6)	24.8 (8.8–67.5)	16.1 (5.2–64.6)	<0.001	0.043
Final GFR (mL/min per 1.73 m^2^)	65 (45–85)	88 (78–104)	70 (59–88)	45 (30–55)	15 (10–21)	<0.001	<0.001
Time to final GFR (years)	4.1 (2.4–7.3)	4.7 (2.5–6.8)	4.0 (2.5–9.0)	4.4 (2.2–6.4)	3.7 (0.9–9.4)	0.797	0.647

G1 - GFR ≥90; G2 - GFR ≥60 and <90; G3 - GFR ≥30 and <60; G4 - GFR ≥15 and <30 mL/min per 1.73 m^2^. Classification based on the best glomerular filtration rate (GFR) achieved up to 18 months after discharge. P1: comparison among all the four groups; P2: comparison between G1+G2 and G3+G4 grouped.

**Figure 3:**
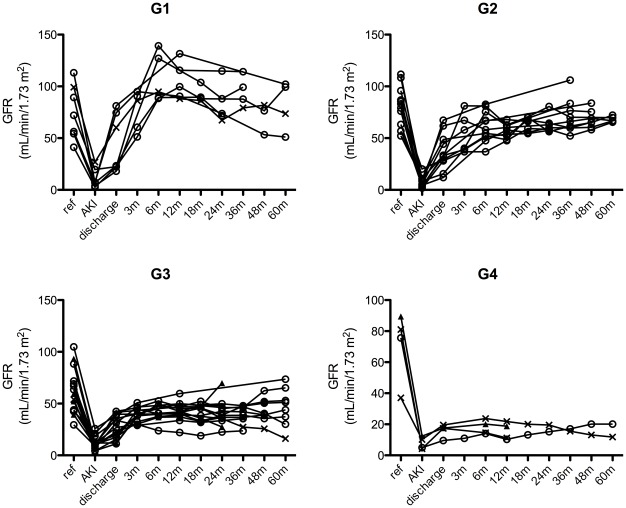
Comparison between reference and best GFR up to 18 months in all groups. GFR: estimated glomerular filtration rate.

Reference GFR was identified for 37 patients with different frequency among the groups:7 from G1 (27%), 11 from G2 (39%), 15 from G3 (62%) and 4 from G4 (67%, p = 0.04) but its value was similar in the 4 groups (P = 0.151). However when G1/G2 was compared to G3/G4, the reference GFR of the first group was higher than the second, respectively 79±22 and 63±21 mL/min per 1.73 m^2^, P = 0.034. Among these 37 patients, we observed that only 2 had recovered renal function at discharge (one from G1 and one from G3). However, by 18 months all patients from G1, 4 from G2 (36%), 5 from G3 (33%) and none from G4 had recovered (p = 0.004). At the time of the best GFR, the number of patients that recovered renal function increased to 6 in G2 (54%) and to 7 in G3 (46%), and the rate of recovery remained different among the 4 groups: P = 0.001. The comparison between reference GFR and best GFR up to 18 months in all groups is shown in figure 3.

The status of the patients in July 2010 is presented in table 4. Patients classified as referred or abandoned lost the follow up in our hospital, thus their actual outcome in July 2010 could not be accessed in the data bank of the hospital. During the follow-up, 6 deaths occurred (7% of the patients). Four patients progressed to dialysis (4.8%), one from G1, one from G3 and two from G4. The patients who died had shorter follow-up [20 months (12–33)] than those who were active [60 months (38–103), P<0.01], than those who had abandoned [45 months (29–96), P<0.05] and those who were referred, [66 months (32–118), P<0.01] but their length of follow-up was similar to those who had progressed to ESRD [62 months (30–74), P>0.05].

**Table 4:** Status of the patients in July 2010.

	All N = 84	G1 N = 26	G2 N = 28	G3 N = 24	G4 N = 6
Active	26 (31%)	3 (12%)	12 (42%)	8 (33%)	3 (50%)
Abandoned	30 (36%)	16 (61%)	11 (39%)	3 (13%)	0
Referred	18 (21%)	5 (19%)	5 (19%)	8 (33%)	0
Dialysis	4 (5%)	1 (4%)	0	1 (4%)	2 (33%)
Death	6 (7%)	1 (4%)	0	4 (17%)	1 (17%)

G1 - GFR ≥90; G2 - GFR ≥60 and <90; G3 - GFR ≥30 and <60; G4 - GFR ≥15 and <30 mL/min per 1.73 m^2^. Classification based on the best glomerular filtration rate (GFR) achieved up to 18 months after discharge.

The final model of the logistic regression is shown in table 5 and was adjusted for diabetes, hypertension, dialysis and nephrotoxicity and hypovolemia etiologies. The Hosmer-Lemeshow test for this model gave a P-value  = 0.853. The risk factors to achieve partial recovery (to be in G3 or G4) after AKI were age [1.09 (1.04–1.14), p<0.0001] and sCr at hospital discharge [2.48 (1.28–4.80, p = 0.007].

**Table 5:** Multiple variable logistic regression for being in G3 or G4 (reference: G1 and G2 grouped).

	OR	CI	P
Age (years)	1.092	1.041–1.146	<0.0001
sCr at hospital discharge (mg/dL)	2.461	1.282–4.727	0.007

The final model was adjusted for the following variables: diabetes, hypertension, dialysis and nephrotoxicity and hypovolemia etiologies. Hosmer-Lemenshow test had a P-value  = 0.853. G1 - GFR ≥90; G2 - GFR ≥60 and <90; G3 - GFR ≥30 and <60; G4 - GFR ≥15 and <30 mL/min per 1.73 m^2^. Classification based on the best glomerular filtration rate (GFR) achieved up to 18 months after discharge. sCr : serum creatinine; OR: odds ratio; CI: 95% confidence interval.

## Discussion

Renal recovery is usually evaluated at hospital discharge and the most frequent definitions are the weaning from dialysis, normalization of sCr or return to sCr baseline value. These criteria are associated with different rates of renal recovery depending on the cohort being evaluated. Studies including only dialyzed patients and considering recovery as weaning from dialysis can show higher rates of recovery than those evaluating patients with AKI based on baseline sCr. (22)Also, studies using estimated baseline sCr can overestimate the incidence of AKI and consequently renal recovery [18]. In addition, most of the studies left the ultimate decision to stop dialysis at the discretion of the treating physician, creating an outcome that is strongly influenced by individual physician and center practice patterns. These disparities in renal recovery definition are reflected on the different rates of reported renal recovery: Bhandari et al found a rate of non-recovery (define as sCr >6.8 mg/dL or requirement of dialysis at 90 days after discharge) from 5% in 1984–1986 to 22% in 1993–1995 [23]. In a more recent review, Bagshaw reported a renal recovery (independence of dialysis at hospital discharge) from 70 to 100% in critically ill patients with AKI requiring dialysis [24].

Within 18 months after AKI, 36% of our patients had GFR <60 mL/min per 1.73 m^2^, but this percentage had been 80% at hospital discharge. Ali et al, in a population-based study, found that among those patients with normal sCr before AKI, 92% had a full recovery (sCr returned to baseline value), 7% had partial recovery (sCr returned to values above the baseline) and only 0.6% maintained dialysis dependence up to 90 days after discharge. Among those patients with abnormal baseline sCr, 65% had full recovery, 29% partial recovery and 6% no recovery [1]. Our study showed a reference GFR higher in G1/G2 than in G3/G4 [79±22 and 63±21 mL/min per 1.73 m^2^, p = 0.03], and an abnormal baseline renal function was associated with a worse renal recovery, similar to the results reported by Ali et al. However, Ponte et al, analyzing 177 survivors of presumed acute tubular necrosis (35% with baseline GFR ≤60 mL/min per 1.73 m^2^), showed a contrasting finding: at discharge, only 26% of those patients without renal dysfunction had returned to the baseline stage of GFR whereas 61% of those with previous renal dysfunction had returned [25].

Advancing from the baseline Kidney Disease Outcomes Quality Initiative (KDOQI) stage to a worse stage is an indication of partial recovery, both for those with or without previous renal dysfunction. Ponte et al, after a median follow-up of 8 years, reported that half of the patients maintained the same KDOQI stage as at hospital discharge. However, the percentage of patients with worsening of KDOQI stage was higher in patients with baseline GFR ≤60 mL/min per 1.73 m^2^ (63.4% versus 37.0%) [25]. Following a cohort of individuals older than 65 years, Sesso et al found an association between the rate of decline in GFR with age and baseline GFR [26]. Similar results were reported by Collins et al [16]. This finding may indicate a mistake in evaluation of the baseline renal function, or it is possible that the patient had a baseline condition leading to GFR decrease, and this condition reverted during the AKI hospitalization. This last hypothesis can explain the findings showed in figure 3 where we can see that many patients in group G1 improved their GFR relative to reference GFR.

The issues of defining AKI based on changes in baseline sCr_,_ can be extended to the definition of recovery [18], [27]. Many parameters have been used to define renal function recovery: independence from dialysis, return to baseline sCr or to baseline GFR, or different levels of sCr. We preferred to define renal recovery as the return to reference GFR instead of to sCr_,_ because using GFR we can classify the patients according to KDOQI stage of chronic disease. However, this choice also presented issues: only 37 of our patients had a known reference sCr and so we could correctly define functional recovery only for them. Moreover, the percentage of patients with known reference sCr was much different among the groups: 33% in G1/G2 but 63% in G3/G4.

Another issue is determining at what point renal recovery must be evaluated: at hospital discharge, after 90 days, after 1 year, etc. Schmitt et al asserted that evaluating renal recovery at discharge is not effective, especially for patients older than 65 years [28]. Our study, which included only patients with little co-morbidity and discharged without requiring dialysis, showed that the median time to reach their highest GFR was one year. However, by one year 14 of our patients were still improving GFR, which stabilized only by 18 months, guiding our decision to evaluate renal recovery by 18 months instead of 12 months year. Additionally, there was a significant range in times to achieve higher GFR, and the patients with best renal function recovery had the shortest time: less than 6 months. Thus, at 6 months or discharge, it is not possible to be confident in estimating GFR by sCr in a patient who had such a severe and catabolic disease as AKI. The loss of muscular mass will show a deceptively low sCr and so will be unreliable to be used as an estimate of GFR. In support of this hypothesis, in G1 the patients had a rapid increase in GFR in the first 6 months after the event and thereafter showed a decline in GFR (figure 2). We can speculate that the recovery of GFR occurred faster than the recovery of muscular mass. Our study suggests that the best parameter to evaluate renal recovery is GFR at 18 months after AKI, as compared to the reference GFR. Collins et al also suggest that renal recovery must be defined relative to baseline GFR [29]. Evaluation at one year after AKI is also a viable option since most of patients who had AKI will likely have sCr tested by that time, regardless of the type of doctor who is attending him or her [16].

The pattern of renal recovery described in our study differs from that described by Liaño et al [30]. They studied 177 patients who survived an AKI episode with a median follow-up of 7 years, and found that sCr at hospital discharge was 1.7±0.7 mg/dL and that it decreased to 1.3±0.5 mg/dL by the 6^th^ month, remaining stable afterwards. During the follow-up, 3 patients (1.7%) required chronic dialysis from 6 to 12 years after hospital discharge. Among the 58 patients more deeply investigated after a median follow-up of 10 years, 79% of them had sCr <1.4 mg/dL, 15% had sCr >1.4 but ≤2 mg/dL, 3% had sCr >2 but ≤4 mg/dL, and only one patient (1.7%) was on chronic dialysis. Our results likely differ due to their larger sample and longer follow-up. Our patients were referred by their attending physician and probably had more severe AKI than those not referred.

It is critical to identify the factors associated with partial renal recovery. Our data showed that only age and sCr at hospital discharge were independent risk factors associated with partial recovery. Schiffl did not find any factor associated with partial recovery (defined as sCr >1.3 mg/dL at hospital discharge) when studying 226 patients with previous normal renal function who had AKI requiring dialysis [22]. James et al studying patients after coronary angiography, observed that those patients who had AKI were more prone to non-renal recovery beyond 3 months after the angiography and more likely to progress toward ESRD. These events were associated with the severity of AKI [31]. However in our study, the peak sCr or the need for dialysis (both indicative of severity of AKI) were not independent factors associated with partial recovery in the logistic regression.

The US Renal Data System 2010 Annual Data Report indicates that the survivors of AKI are at risk of developing ESRD within the following year. This risk increases from less than 1% for those without previous CKD to 5% for those with previous CKD [16]. Our data indicated that 4 patients (4.7%) have progressed to ESRD, however, only one (1.1%) within the first year after AKI. It is uncertain whether our nephrology care provided better prognosis to these patients or if our patients had less co-morbidities than those evaluated by the US Renal Data System. In patients with diabetes and CKD nephrologic care decreased mortality [32]. Since 48 patients of our patients (57%) abandoned the follow up, we cannot be sure about the actual number of patients who progressed to ESRD.

Our study has several limitations and cannot be generalized for patients with AKI as a whole. It is one-center retrospective study; the intervals for GFR testing were determined by the clinical conditions of each patient and not pre-specified. Only 44% of our patients had known reference sCr, and so the renal recovery based on return of GFR to baseline could be evaluated only for that 44%. It is possible that more patients without measured reference sCr were in G1 and G2 as they were younger and healthier. We did not define AKI by RIFLE or AKIN criteria, but all patients had severe AKI (peak sCr ≥3 mg/dL) and the same AKI definition was adopted throughout the entire study. Also, our patients were referred to the nephrologist because they had an unusual pattern of AKI in some way and only 22 patients (26%) had surgical AKI.

The results of our study are applicable mainly to patients with acute tubular necrosis, the commonest cause of AKI in hospital: 94% of our patients had this presumed cause. The follow-up was made by a single nephrologist, who was well aware of the possibility of AKI progression to CKD and tried to preserve renal function. Thus, our results might be indicating a better than usual long-term outcome after AKI: only 4.7% of progression to ESRD after a median time of 4.7 years. However, we could not definitely determine the outcome in 30 patients (35%) who abandoned the follow-up at our hospital. Notwithstanding, the follow-up of these patients was similar to the others, except for those who died and who had shorter follow-up.

In conclusion, the pattern of renal recovery after AKI could be described as follows: recovery varies in the patients and its achievement is better evaluated after at least 1 year after the hospital discharge. Facilitating a more functional recovery is a subject that requires further studies, but the survivors of AKI deserve a careful and long-term medical follow-up.
